# Computational Evaluation of Statin Analogs Targeting HMG‐CoA Reductase for Coronary Artery Disease Treatment

**DOI:** 10.1002/open.202500533

**Published:** 2025-11-10

**Authors:** Yoshua B. Mtulo, Geradius Deogratias, James E. Mgaya, Andrew S. Paluch, Lucas Paul

**Affiliations:** ^1^ Department of Chemistry Dar es Salaam University College of Education Dar es Salaam Tanzania; ^2^ Chemistry Department College of Natural and Applied Sciences University of Dar es Salaam Dar es Salaam Tanzania; ^3^ Department of Chemical, Paper, and Biomedical Engineering Miami University Oxford Ohio USA

**Keywords:** ADMET, cardiovascular disease, docking, HMG‐CoA, molecular dynamics simulation, statins

## Abstract

Cardiovascular diseases remain a leading cause of global mortality. While statins are pivotal in managing risk, most research focuses on their derivatives. This study provides a novel computational evaluation of statin analogs, addressing a significant literature gap. Our comprehensive in silico approach, integrating ADMET profiling, molecular docking, and extensive 200‐ns molecular dynamics (MD) simulations, investigated the pharmacokinetic behavior, binding affinities, and structural stability of five statin analogs against HMG‐CoA reductase. ADMET analysis showed that analogs of simvastatin, lovastatin, and pravastatin have favorable pharmacological profiles and low toxicity. While docking showed that simvastatin and lovastatin analogs had the strongest affinities, MD offered critical mechanistic insights. The unbound enzyme exhibited significant conformational flexibility. In contrast, binding induced a superior stabilizing effect, confining the protein to a single, compact, low‐energy state, as confirmed by free energy landscape analysis. This ligand‐induced rigidity is a powerful indicator of enhanced inhibitory efficacy and stability. Our findings highlight that statin analogs are a promising class whose unique binding dynamics offer a new, rational pathway for designing more effective HMG‐CoA reductase inhibitors.

## Graphical abstract

Molecular dynamics analysis of novel statin analogs shows that binding induces superior stabilization of HMG‐CoA reductase. As shown by the solvent‐accessible surface area profile, ligand‐induced rigidity offers a new, effective strategy for drug design.

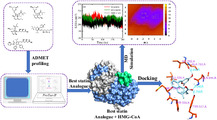

## 1 Introduction

Cardiovascular disease (CVD) encompasses a range of disorders that affect the heart and blood vessels, including coronary artery disease (CAD), heart failure, arrhythmias, and stroke [1]. It remains the foremost cause of death globally, posing a significant public health challenge [2].

Among the various forms of CVD, CAD, which results from cholesterol plaque accumulation within coronary arteries, is the most common and fatal, causing nearly 9 million deaths annually [3]. The global impact of CAD remains high, underscoring the urgent need for effective prevention and treatment strategies. Encouragingly, up to 90% of CAD cases are considered preventable through lifestyle interventions [4]. These include regular physical activity, dietary changes, smoking cessation, and pharmacological treatments where necessary [5]. However, many of these pharmacological treatments cause side effects like myopathy and hepatotoxicity, especially with long‐term use [6]. These limitations have led to increased interest in safer and biocompatible alternatives, including natural and semisynthetic compounds.

**FIGURE 1:**
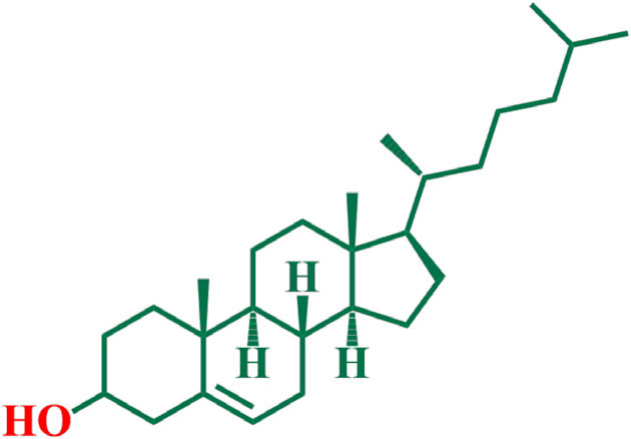
Chemical structure of cholesterol.

**FIGURE 2:**
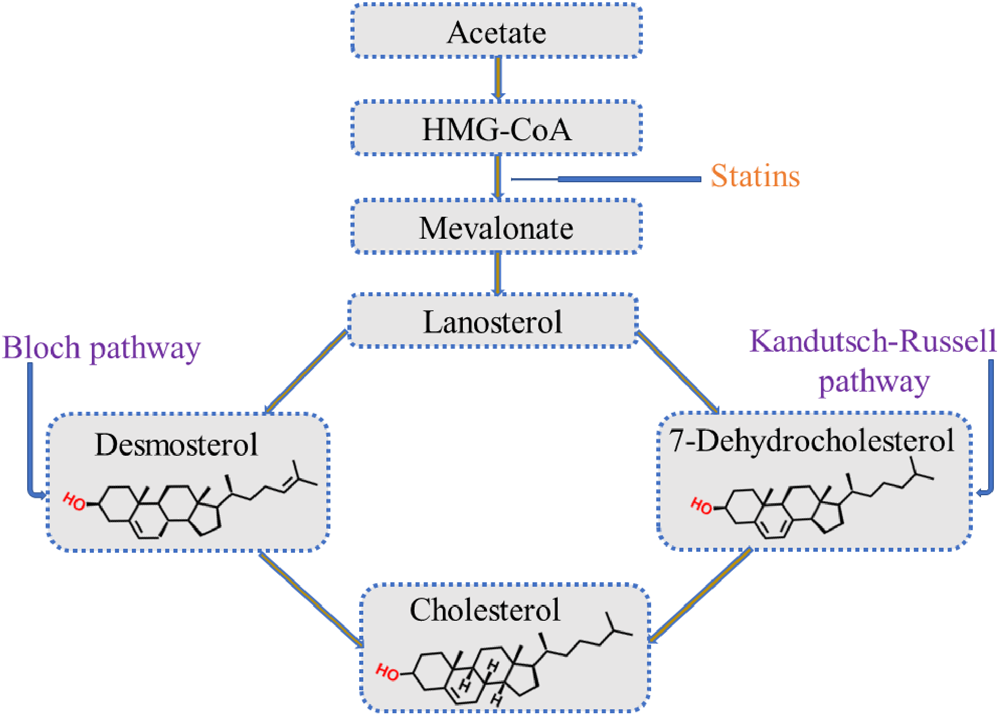
Mechanism of cholesterol synthesis.

Given their central role in reducing low density lipoprotein (LDL) cholesterol (Figure 1), statins remain the first‐line pharmacological treatment for CAD [7]. Statins target 3‐hydroxy‐3‐methylglutaryl‐coenzyme‐A (HMG‐CoA) reductase, the key regulatory enzyme in the mevalonate pathway (Figure 2), by acting as competitive inhibitors [8]. By blocking the reduction of HMG‐CoA to mevalonate, statins reduce cholesterol production and induce the upregulation of LDL receptors, enhancing the clearance of circulating LDL cholesterol [9]. Nevertheless, several clinically approved statins (derivatives) are associated with limitations such as low aqueous solubility and suboptimal pharmacokinetic profiles [10].

**FIGURE 3:**
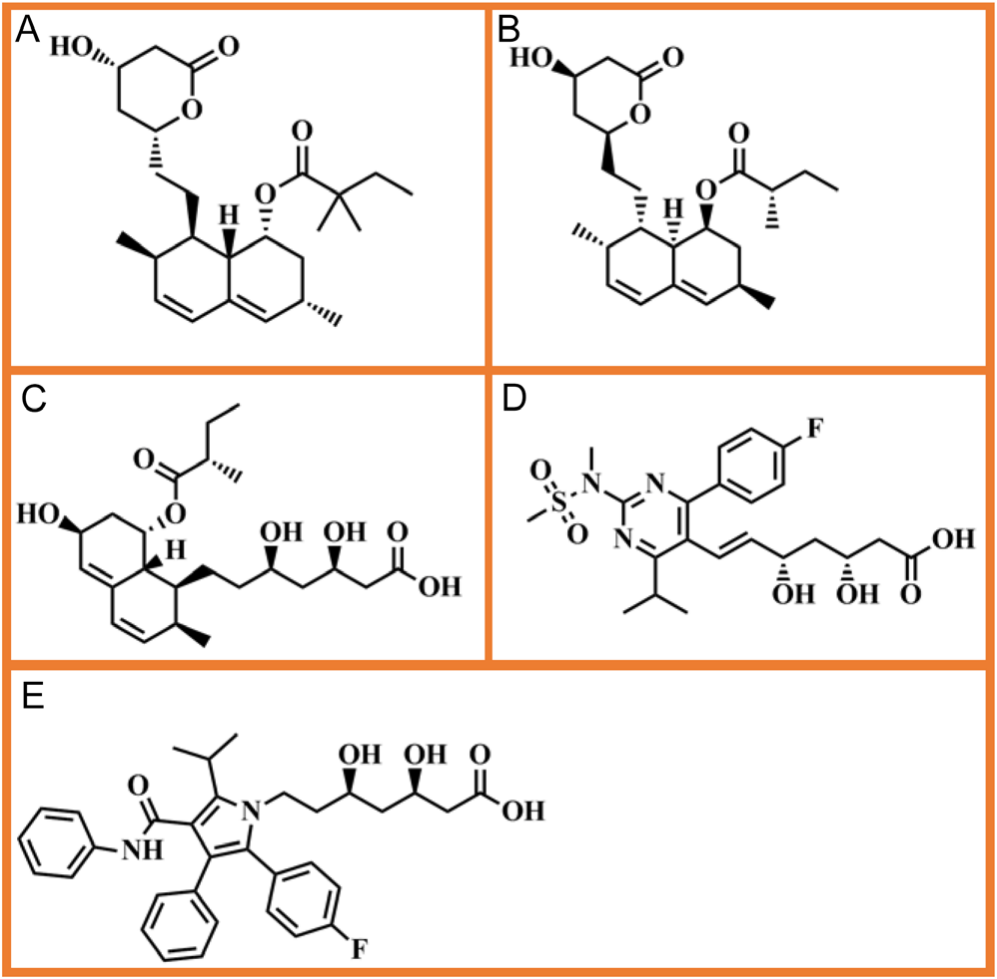
Statins derivatives chemical structures where (A) simvastatin, (B) lovastatin, (C) pravastatin, (D) rosuvastatin, and (E) atorvastatin.

A key consideration in rational drug design is the exploration of new molecular scaffolds that can enhance therapeutic efficacy. While existing research has focused on minor modifications to the core statin structure (derivatives) (Figure 3), the potential of structurally distinct statin analogs has been largely overlooked. Analogs, which retain the core pharmacophore but differ in their peripheral substituents, offer a unique opportunity to design inhibitors with improved stability and therapeutic profiles.

Therefore, this study aims to comprehensively evaluate selected statin analogs using an advanced in silico framework. Our objective is not merely to screen for new drug candidates but to provide a deeper, mechanistic understanding of how these distinct analogs interact with HMG‐CoA reductase at the atomic level. We use absorption, distribution, metabolism, excretion and toxicity (ADMET) profiling, molecular docking to identify promising candidates, and most importantly, extensive molecular dynamics (MD) simulations and free energy landscape (FEL) analysis to validate and gain insight into their therapeutic potential. This robust, multistage approach moves beyond the routine application of docking by using MD to provide a genuinely new insight into how these compounds stabilize the enzyme. Our evaluation is designed to identify optimized analogs that induce superior structural stabilization and rigidity in the target enzyme, thereby offering a new avenue for the development of more effective and durable treatments for CAD.

## 2 Methodology

### 2.1 Ligand and Protein Preparation

The statin analogs were obtained from the PubChem database [11] in SDF format. Energy minimization was performed in Open Babel [12] using the Merck Molecular Force Field 94 (MMFF94) [13] for 3,000 steps. The minimized structures were then saved in the Protein Data Bank, partial charges and torsions (PDBQT) format [14], which is commonly used in molecular docking simulations. The three‐dimensional structure of the target protein, HMG‐CoA reductase, was obtained from the Protein Data Bank [15] (PDB ID: 1HW9) [16]. The crystal structure consisted of four chains and was cocrystallized with simvastatin ligand. UCSF Chimera [17] was used to remove all chains except chain A. Additionally, hydrogen atoms were added and appropriate charges were assigned to the protein to ensure accurate geometry and electrostatic interactions, which are critical for reliable docking results [18]. The protein was then converted into the PDBQT format [14], which includes specific atom types suitable for molecular docking procedures.

### 2.2 ADMET Profiling

The pharmacokinetic and safety profile of a drug candidate plays a crucial role in assessing its suitability for therapeutic use during the drug development process [19]. To evaluate pharmacokinetic behavior and safety, ADMET analysis was conducted using SwissADME [20] and ProTox‐III [21] tools. SwissADME [20] was used to predict key parameters such as gastrointestinal absorption, lipophilicity (LogP), molecular weight, blood‐brain barrier permeability, and adherence to Lipinski's rule of five. The platform also assessed the potential of the compounds to inhibit major cytochrome P450 (CYP450) enzymes, which are critical in drug metabolism. To evaluate safety profiles, ProTox‐III [21] was employed to predict acute toxicity (LD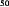). LD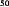 refers to the lethal dose required to cause death in 50% of a test population and is commonly used as an indicator of toxicity level of a compound [22]. Additional predictions included hepatotoxicity, carcinogenicity, immunotoxicity, and cytotoxicity which was analyzed by ProTox‐III [21]. Compounds that showed favorable pharmacokinetic characteristics, minimal cytochrome P450 inhibition and low toxicity risk were selected for further molecular docking and MD simulations.

### 2.3 Docking

Molecular docking is a computational technique used to predict how small molecules, such as drugs, interact with a target protein at the atomic level [23]. In this study, docking experiments were conducted using AutoDock Vina [24] to evaluate the binding potential of statin analogs to the target protein.

Blind docking was intentionally employed to enable an unbiased exploration of all potential ligand‐binding regions across the protein surface. This approach was particularly chosen to assess whether statin analogs could identify alternative or secondary binding pockets beyond the known cocrystallized active site (PDB ID: 1HW9).

A sufficiently large grid box (x=y=z=126 Å) was therefore defined to cover the entire receptor structure, allowing the ligand to freely move around the whole protein and search for the most favorable active site. Although such a grid dimension is computationally demanding, it ensures comprehensive coverage of possible binding regions and provides insight into ligand accessibility and conformational adaptability of the protein.

The exhaustiveness parameter was set to 8 to ensure thorough conformational sampling [25]. During the docking process, ligands were treated as flexible, while the receptor was kept rigid [26]. Key interactions, including hydrogen bonds and hydrophobic contacts, were further analyzed using UCSF Chimera [17] to understand the molecular basis of binding.

### 2.4 MD Simulation

MD simulation is a computational method that numerically integrates the classical equations of motion to predict the time‐dependent behavior of atoms and molecules, thereby enabling the investigation of structural, dynamical, and thermodynamic properties of matter at the atomic scale [27]. MD helps in understanding the stability, flexibility, and interaction patterns of biomolecular systems. MD simulations were performed using the GROMACS 2018.1 [28] software package to examine the interaction behavior and structural stability of HMG‐CoA reductase in complex with selected statin analogs. The simulation was set up using the protein‐ligand docking conformations that showed the highest scores. The bonded and nonbonded interactions were parameterized using the OPLS‐AA force field [29], and ligand parameters were generated using the LigParGen server [30]. The complexes were solvated in a cubic box with the TIP4P water model [31]. Both sodium ( ${\mathrm{Na}}^{+}$) and chloride ( ${\mathrm{Cl}}^{-}$) ions were added to neutralize the system and maintain electrostatic stability under periodic boundary conditions. Adding these ions is important to mimic physiological ionic strength and ensure realistic simulation conditions [32]. Energy minimization was conducted using the steepest descent algorithm to remove steric clashes and optimize geometry [33]. During the initial equilibration, positional restraints were applied to the heavy atoms of both protein and ligand to prevent significant structural distortions. The system was first equilibrated under constant number of particles, volume, and temperature [34] conditions for 1 ns using the stochastic velocity‐rescaling thermostat (v‐rescale) at 300 K [35]. This was followed by constant number of particles, pressure, and temperature [36] equilibration for 1 ns using the Parrinello Rahman barostat [37] at 1 bar pressure. A 200 ns production simulation was then performed under stable temperature and pressure conditions using the same thermostat and barostat settings. Long‐range electrostatic interactions were treated using the particle mesh Ewald [38] method with a cutoff of 1.0 nm for both Coulomb and van der Waals interactions. Bond constraints involving hydrogen atoms were managed using the LINCS algorithm [39] and the simulation was run with a 2 fs time step, saving the trajectory every 10 ps. Trajectory analyses included the computation of root mean square deviation (RMSD), root mean square fluctuation (RMSF), radius of gyration (Rg), and solvent‐accessible surface area (SASA) to monitor system stability and conformational changes. Additionally, FEL analysis was applied.

#### 2.4.1 FEL Analysis

To better understand the conformational behavior of the protein systems, a FEL analysis was conducted following the MD simulations.

The FELs were constructed based on the first two principal components obtained from the principal component analysis (PCA), which captures the dominant motions of the protein over the course of the simulation. Consistent with the PCA procedure, the FEL analysis was performed using the Cα atomic coordinates, which effectively describe the collective backbone fluctuations. The trajectory files from the 200 ns MD simulations were first preprocessed by removing periodic boundary conditions and aligning the structures to the reference frame to eliminate translational and rotational motions.

Trajectory frames were extracted every 10 ps, resulting in 20,000 frames analyzed for each system to ensure adequate sampling of conformational space. Covariance matrices were then generated using the backbone atoms of the protein, and eigenvectors and eigenvalues [40] were calculated to extract principal components.

The first and second principal components (PC1 and PC2) were plotted against each other to visualize the conformational space sampled during the simulation. The free energy corresponding to each conformation was calculated using the standard expression in Equation (1) [41]

(1) ΔG=−RTln P

where ΔG is the free energy, R is the universal gas constant, T is the absolute temperature in Kelvin, and P is the probability of a given conformation occurring in the trajectory.

The resulting two‐dimensional FEL maps represent the distribution of conformational states along PC1 and PC2, with energy basins corresponding to stable structural arrangements. These maps were used to evaluate the impact of ligand binding on the conformational stability and energy minima of HMG‐CoA reductase, revealing distinct stability basins between the apo protein and its statin‐bound complexes.

## 3 Results and Discussion

### 3.1 ADMET Properties Prediction of Statin Analogs

ADMET is a fundamental aspect of drug discovery that helps in evaluating pharmacokinetics and safety [42]. These properties are essential for identifying compounds with desirable bioavailability, minimal toxicity, and strong therapeutic potential [43]. In this study, the selected statin analogs were assessed for their drug‐likeness, pharmacokinetic behavior, and toxicity to support their suitability as potential drug candidates.

#### 3.1.1 Drug‐Likeness Properties

The majority of analogs exhibited favorable drug‐likeness characteristics by conforming to Lipinski's rule of five (Table 1). All the analogs have molecular weights under 500 Da, meeting the criteria of Lipinski's rule of five [44], with the exception of atorvastatin analogs, which exceeded the limit at 558.64 Da. This suggests potential limitations in permeability and systemic absorption. LogP values ranged from 2.28 to 4.94, falling within acceptable boundaries for oral drugs [45]. Simvastatin, lovastatin, and pravastatin analogs exhibited optimal lipophilicity (LogP values of 4.10, 3.83, and 2.43, respectively), low molecular weight, and good hydrogen bonding capacity, which are all consistent with high gastrointestinal absorption, this is consistent with the findings reported by Ursu [45]. These findings highlight why some analogs, such as atorvastatin, may face absorption challenges, whereas others demonstrate profiles more consistent with good oral bioavailability.

**TABLE 1:** Drug‐likeness predictions for the proposed statin derivatives as predicted by SwissADME [20]. All derivatives showed no PAINS  alerts (score = 0).

Mol	Simv	Ato	Lov	Prav	Rosu
MW	418.57	558.64	404.54	424.53	481.54
LogP	4.10	4.94	3.83	2.43	2.28
NR	7	13	7	11	10
HBA	5	6	5	7	9
HBD	1	4	1	4	3
TPSA	72.83	111.79	72.83	124.29	149.30
Bio S.	0.55	0.56	0.55	0.56	0.56
Syn A.	5.80	4.95	5.76	5.88	4.60

**Key:** PAINS = pan assay interference compounds alert, Mol = molecule, Simv = simvastatin, Ato = storvastatin, Lov =  lovastatin, Prav = pravastatin, Rosu = rosuvastatin, MW = molecular weight, LogP = octanol/water partition coefficient, NR = number of rotatable bonds (single nonring bonds, excluding bonds to H and aromatic rings; calculated using SwissADME [20]), HBA = hydrogen bond acceptors, HBD = hydrogen bond donors, TPSA = topological polar surface area (nm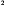), Bio S. = bioavailability ccore, Syn A. = synthetic accessibility.

Topological polar surface area (TPSA) values further supported favorable absorption, with simvastatin and lovastatin analogs both recording TPSA of 72.83 nm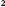, while pravastatin analogs had a slightly higher value of 124.29 nm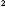. Bioavailability scores ranged from 0.55 to 0.56 for all analogs, and synthetic accessibility scores were also within an acceptable range (4.60–5.88), indicating that the analogs are synthetically feasible. Importantly, none of the analogs triggered pan assay interference compounds alert (PAINS) alerts, highlighting their structural suitability for drug development. Generally, these results fall within the drug‐likeness range as reported in the study by Elharafi [46].

#### 3.1.2 Pharmacokinetics Properties

The pharmacokinetic profile indicated that most analogs exhibited high gastrointestinal absorption, particularly simvastatin, lovastatin, and pravastatin (Table 2). In contrast, atorvastatin and rosuvastatin analogs showed lower absorption, likely due to their higher molecular weights and TPSA, which can hinder membrane permeability [45]. None of the analogs were predicted to cross the blood‐brain barrier, with the exception of lovastatin, which displayed potential permeability, likely related to its optimal balance of lipophilicity and molecular size, these findings agree with those reported in the study by Sierra et al. [47].

**TABLE 2:** Pharmacokinetics predictions for the proposed statin derivatives. All derivatives exhibited moderate solubility and did not show any inhibition of CYP1A2 [20].

Molecule	Simv	Ato	Lov	Prav	Rosu
GI absorption	High	Low	High	High	Low
BBB permeant	No	No	Yes	No	No
Pgp substrate	No	Yes	No	Yes	Yes
CYP2C19 Inh	No	Yes	No	No	No
CYP2C9 Inh	Yes	No	Yes	No	No
CYP2D6 Inh	No	Yes	No	No	No
CYP3A4 Inh	Yes	Yes	Yes	Yes	No
log Kp (cm/s)	−5.53	−6.19	−5.74	−7.12	−8.07

**Key:** BBB = blood‐brain barrier, Pgp = P‐glycoprotein, log Kp = skin permeability in cm/s, and Inh = Inhibitor.

Pravastatin, atorvastatin, and rosuvastatin analogs were predicted to be P‐glycoprotein substrates meaning that they are likely subject to efflux activity, which may affect their retention at target sites [48]. Regarding cytochrome inhibition, simvastatin and rosuvastatin inhibited CYP3A4 and CYP2C9 selectively, while showing no inhibition of CYP1A2, CYP2C19, and CYP2D6. Atorvastatin showed inhibition of CYP2C19, CYP2D6, and CYP3A4, but not CYP1A2 or CYP2C9. Lovastatin inhibited CYP2C9 and CYP3A4, while pravastatin exhibited no inhibitory activity against any of the cytochrome enzymes, underscoring its metabolic compatibility. These results indicate that all analogs do not inhibit certain cytochrome enzymes, which helps to prevent metabolic interaction and support safe co‐administration with other drugs, consistent with the findings reported by Lin and Lu [49].

The skin permeability (log Kp) of the statin analogs ranged from –5.53 cm/s for simvastatin to –8.07 cm/s for rosuvastatin, indicating limited transdermal absorption across all compounds. Simvastatin and lovastatin exhibited relatively higher permeability, whereas rosuvastatin and pravastatin demonstrated the lowest values, likely due to differences in molecular weight, lipophilicity, and hydrogen bonding capacity, which can influence membrane penetration. This suggests minimal potential for transdermal drug delivery [50].

Additionally, pravastatin and rosuvastatin are cleared relatively fast, which might require more frequent dosing, while atorvastatin is eliminated slowly, allowing longer exposure that could increase the risk of adverse effects. These observations are consistent with previous reports [51].

#### 3.1.3 Toxicity Predictions

Toxicity analysis was conducted using the ProTox III [21]. Pravastatin analogs emerged as the safest compounds with an LD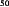 value of 8939 mg/kg and was assigned toxicity class 6, indicating a very low risk of acute toxicity (Table 3). Simvastatin and lovastatin analogs were categorized under class 4 toxicity, both with LD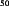 values of 1000 mg/kg, suggesting moderate toxicity levels but still within acceptable therapeutic limits.

**TABLE 3:** Toxicity predictions for selected statin derivatives. All compounds were predicted to have active immunotoxicity, inactive mutagenicity and cytotoxicity [21].

Compound	Simv	Lov	Prav	Rosu
Hepatotoxicity	Inactive	Inactive	Inactive	Active
LD 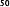 (mg/kg)	1000	1000	8939	464
Toxicity class	4	4	6	4

**Key:** Simv = simvastatin, Lov = lovastatin, Prav = pravastatin, Rosu = rosuvastatin.

In contrast, rosuvastatin analogs displayed the highest risk, with a predicted LD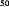 of 464 mg/kg and active hepatotoxicity, placing them in toxicity class 4 [52]. The elevated toxicity is linked to their larger molecular size, higher polarity, and functional groups that interact strongly with liver enzymes. All analogs were predicted to exhibit active immunotoxicity, highlighting the need for further investigation to evaluate potential immune‐related effects, while none were mutagenic or cytotoxic.

Taken together, lovastatin, simvastatin, and pravastatin analogs demonstrated the most favorable ADMET profiles. They combined good oral absorption, low enzyme inhibition, low toxicity, and compliance with drug‐likeness rules. These analogs were therefore selected for further computational studies.

### 3.2 Binding Affinity and Interaction

#### 3.2.1 Molecular Docking

Molecular docking is an essential computational technique for predicting ligand‐receptor binding affinities, thereby supporting rational drug design and optimization of molecular interactions with therapeutic targets [53]. In this study, docking simulations were conducted to assess the interaction potential of statin analogs with HMG‐CoA reductase PDB ID 1HW9. A total of 72 simvastatin, 108 lovastatin, 66 pravastatin, and 8 rosuvastatin analogs obtained from the ZINC database were screened against the target protein. The average binding energies for each statin analog are presented in Table 4, with simvastatin and lovastatin analogs exhibiting the strongest binding energies. This is attributed to their favorable molecular size, optimal lipophilicity, and hydrogen bonding potential, which enhance interactions with the target binding site [10].

**TABLE 4:** Binding energy results for the statin derivatives with HMG‐CoA reductase receptor.

Molecule	Average Binding Energy [kcal/mol]
Simv	−7.56
Lov	−7.58
Prav	−6.80
Rosu	−6.92

**Key:** Simv = simvastatin, Lov = lovastatin, Prav = pravastatin, Rosu = rosuvastatin.

**FIGURE 4:**
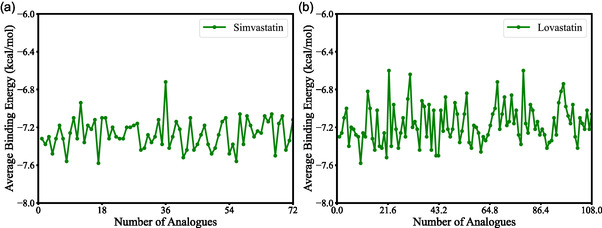
Binding affinity profiles of HMG‐CoA reductase with selected statin analogs based on molecular docking results, where (a) shows the binding energies of 72 simvastatin analogs and (b) displays the binding energies of 108 lovastatin analogs docked with HMG‐CoA reductase.

For simvastatin analogs, binding energies ranged from –6.72 to –7.56 kcal/mol (Figure 4a). The narrow distribution, with most compounds clustering around –7.30 kcal/mol, indicates consistent and stable interactions between these ligands and the enzyme's active site. The analog with the most favorable energy value was predicted to form the most stable complex, suggesting enhanced inhibition potential and greater therapeutic promise.

Lovastatin analogs displayed binding energies between –6.60 and –7.70 kcal/mol (Figure 4b). The substantial proportion of compounds near –7.20 kcal/mol demonstrates a relatively uniform binding profile. The top‐scoring analog, with the most negative binding energy, is expected to form a strong and stable complex with HMG‐CoA reductase, a desirable feature in the development of effective enzyme inhibitors.

From a drug design perspective, more negative binding energy values indicate stronger ligand‐target interactions, which often correlate with higher inhibitory potency [54]. Among the statin analogs, simvastatin (ZINC9231494, –7.56 kcal/mol), and lovastatin (ZINC3833890, –7.70 kcal/mol) exhibited the strongest binding affinities, suggesting their greater potential as lead scaffolds for structural optimization. These analogs were prioritized for subsequent investigations, aiming to evaluate their molecular interactions and dynamic stability, with the goal of identifying promising candidates for improving therapeutic outcomes in CAD treatment.

#### 3.2.2 HMG‐CoA Analogs Interaction

**FIGURE 5:**
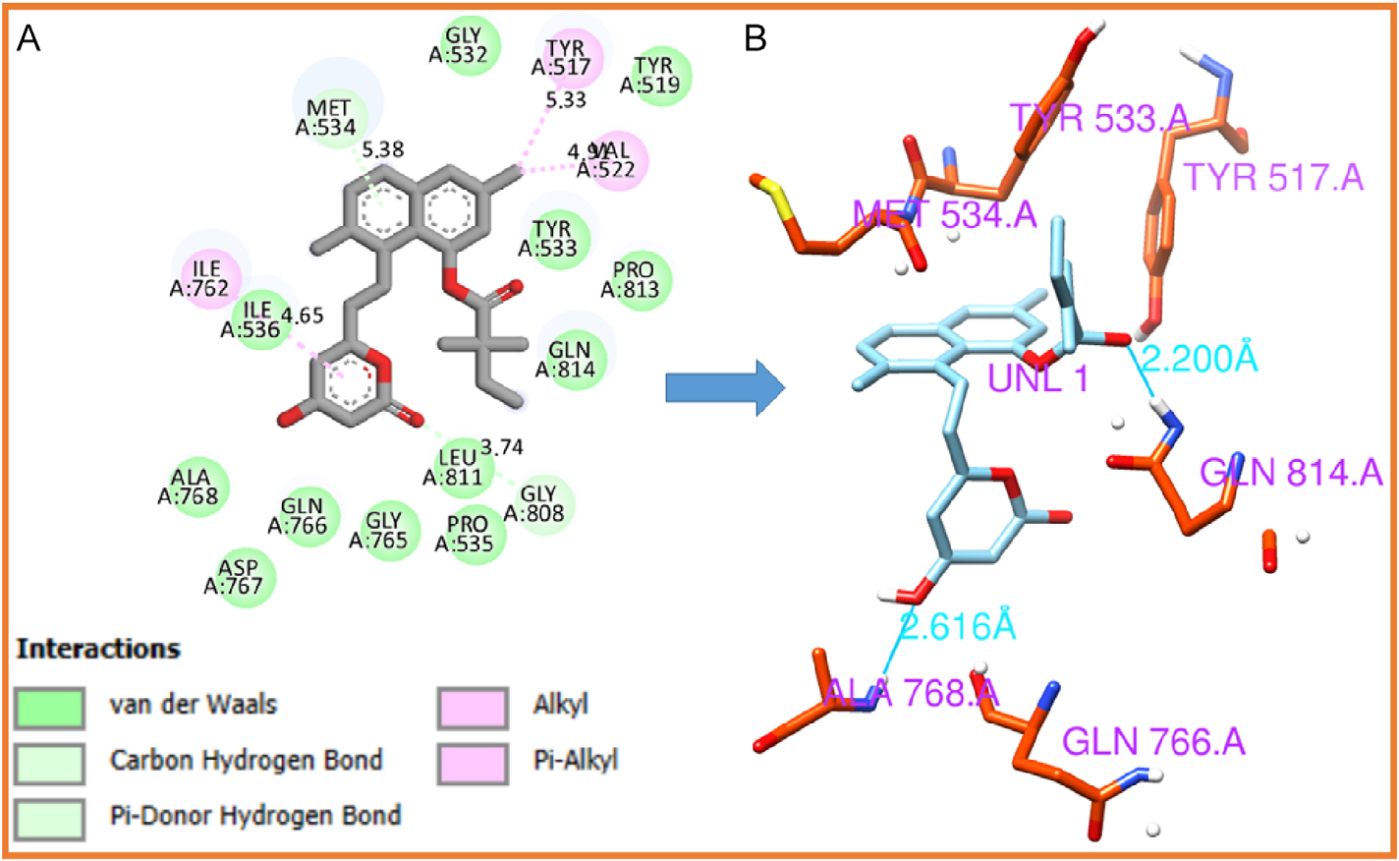
Molecular interactions of simvastatin with HMG‐CoA reductase. (A) Overall binding conformation of simvastatin within the active site of HMG‐CoA reductase, demonstrating key hydrophobic and polar contacts. (B) Detailed view of the hydrogen bonding interactions between simvastatin and catalytic residues of HMG‐CoA reductase, highlighting the molecular basis of ligand stabilization.

**FIGURE 6:**
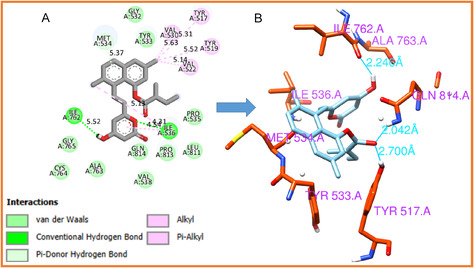
Binding interactions of lovastatin with HMG‐CoA reductase. (A) Three‐dimensional structure of HMG‐CoA reductase in complex with lovastatin, illustrating its orientation within the enzyme's binding pocket. (B) Hydrogen bond network formed between lovastatin and key active‐site residues, supporting the ligand's binding affinity and stabilization.

To gain deeper insight into the molecular interactions underlying the observed binding affinities, the complexes of the top‐ranked ligands were examined in detail. The interaction profiles were visualized using Discovery Studio [55], and the results are presented in Figures 5 and 6. Analysis revealed that simvastatin and lovastatin establish multiple stabilizing contacts with HMG‐CoA reductase, including van der Waals forces, hydrogen bonds, and alkyl interactions with specific amino acid residues, as summarized in Table 5. Notably, the lovastatin complex forms three hydrogen bonds, whereas the simvastatin complex forms two. The presence of multiple hydrogen bonds enhances the specificity and strength of ligand binding, contributing to improved stability of the enzyme–ligand complex. From a drug design perspective, such interactions are crucial because they increase the likelihood of effective target engagement and sustained inhibition of HMG‐CoA reductase. This inhibition interrupts the conversion of HMG‐CoA to mevalonate, a key step in cholesterol biosynthesis, which is clinically important in managing hypercholesterolemia and reducing cardiovascular risk [54].

**TABLE 5:** Key interactions of statin derivatives with HMG‐CoA reductase.

CPD	Alk/Pi‐Alk	H‐bond	VdW
Simv	Tyr517, Val522, Ile762	Gln814, Ala768	Tyr519, Tyr533, Pro813, Leu811, Gly808, Pro535, Gly765, Gln766,
			Asp767, Ile536, Met534, Gly532
Lov	Tyr519, Val530, Val522	Ile762, Gln814, Tyr517	Pro535, Leu811, Pro813, Gln814, Val538, Ala763, Cys764, Gly765,
			Met534, Gly532, Tyr533

**Key:** CPD = compound, Simv = simvastatin, Lov = lovastatin, Alk/Pi‐Alk = alkyl and Pi‐alkyl interactions, H‐bond = hydrogen bond, VdW = van der Waals interactions.

### 3.3 MD Simulation

#### 3.3.1 HMG‐CoA Conformations

Conformational changes were assessed using the RMSD, which measures the displacement of atomic positions relative to the initial structure and serves as an indicator of the stability of the protein‐ligand complex [56].

The average RMSD values for the apo protein and its complexes with simvastatin and lovastatin were 0.599 ± 0.143, 0.396 ± 0.068, and 0.359 ± 0.058 nm, respectively. The higher RMSD value of the unbound form reflects greater structural fluctuations, indicating reduced stability compared to the ligand‐bound complexes. This observation is consistent with the stabilizing effect of ligand binding, where interactions within the active site restrict large‐scale protein movements [57].

**FIGURE 7:**
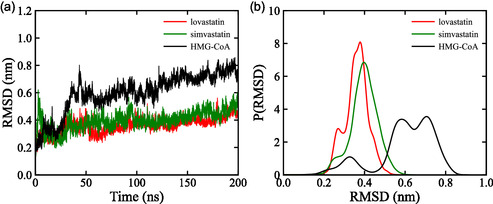
Structural stability of HMG‐CoA reductase analysis where panel (a) displays RMSD profiles of the free protein compared to its complexes with lovastatin and simvastatin, and (b) illustrates the probability density of structural deviations, highlighting conformational differences between the free and ligand‐bound protein forms.

Throughout the simulation, the unbound enzyme displayed an initial period of moderate fluctuations followed by a sharp increase in RMSD at ≈40 ns, reaching a maximum of 0.80 nm. This transient peak suggests a significant conformational rearrangement, after which the structure gradually stabilized. Overall, fluctuations ranged from 0.19 to 0.80 nm, with the majority of values falling between 0.50 and 0.70 nm (Figure 7a). In contrast, both lovastatin‐ and simvastatin‐bound complexes exhibited lower fluctuations during the first 50 ns, after which they maintained stable conformations, indicating improved structural integrity upon ligand association.

The probability density analysis of RMSD values revealed two dominant conformations for the unbound HMG‐CoA reductase, centered at 0.32 and 0.65 nm with probabilities of 0.83 and 3.08, respectively. This bimodal distribution suggests higher conformational flexibility and the existence of multiple structural states in the absence of ligand binding [57]. In comparison, the lovastatin complex and simvastatin complex forms displayed unimodal distributions centered at 0.37 and 0.41 nm, with higher probabilities of 6.79 and 5.89, respectively (Figure 7b). These narrow, high‐probability peaks indicate that ligand binding reduces conformational variability and promotes a more compact and stable protein structure.

From a biochemical perspective, the reduced structural fluctuations in the ligand‐bound forms can be attributed to stabilizing hydrophobic and hydrogen bonding interactions between the statins and key active‐site residues [58]. This stabilization likely enhances the inhibitory efficiency of the compounds by maintaining the active site geometry required for effective enzyme inhibition. The observed trends are in agreement with previous findings by Suganya [59], further supporting the role of statin binding in modulating the conformational dynamics of HMG‐CoA reductase.

#### 3.3.2 SASA

In this study, SASA was evaluated for the apo form of HMG‐CoA reductase, the HMG‐CoA reductase complex with lovastatin, and the HMG‐CoA reductase complex with simvastatin over a 200 ns MD simulation. The average SASA values for apo protein and its complexes with simvastatin and lovastatin were 205.229 ± 2.486, 206.392 ± 2.918, and 206.447 ± 4.071 nm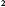, respectively.

**FIGURE 8:**
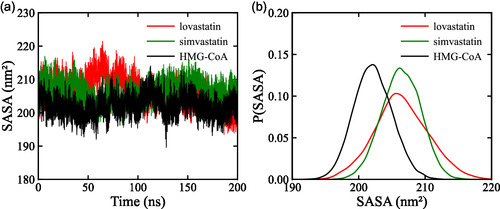
Solvent exposure and structural variation of HMG‐CoA reductase, where panel (a) shows SASA values for the apo enzyme and its complexes with lovastatin and simvastatin, and panel (b) presents the probability density of SASA values, comparing the structural behavior of the apo enzyme and the ligand‐associated forms.

As shown in Figure 8a, the apo protein maintained a relatively compact conformation with SASA fluctuations centered around 200 nm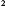. In comparison, both statin complexes showed a modest increase in SASA, with the complex containing simvastatin displaying the highest average values, ≈210 nm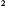. This suggests that ligand binding may increase solvent exposure near the active site, possibly reflecting local relaxation or flexibility [60].

The SASA probability density presented in Figure 8b revealed distinct conformational states. The apo enzyme displayed a peak probability of 0.12 at 202.14 nm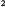, indicating its compact structural state. For the complex with lovastatin, the peak shifted to 206.16 nm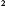 with a probability of 0.09, indicating moderate expansion. The complex with simvastatin exhibited a peak probability of 0.12 at 206.53 nm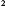, corresponding to greater solvent exposure compared with both the apo enzyme and the lovastatin complex.

These findings indicate that the binding of statins induces slight conformational rearrangements in HMG‐CoA reductase. The slightly higher SASA observed in the complex with simvastatin is consistent with increased local flexibility and solvent exposure, potentially affecting the thermodynamic landscape of ligand binding [61].

#### 3.3.3 Analysis of Radius of Gyration (Rg) for HMG‐CoA Reductase

**FIGURE 9:**
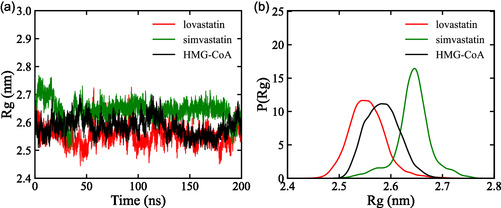
Radius of gyration (Rg) analysis of HMG‐CoA reductase during 200 ns simulation. (a) Rg profiles of the apo protein and its complexes with lovastatin and simvastatin. (b) Probability density of Rg values for each system, illustrating differences in structural compactness and flexibility.

Overall compactness and conformational stability of proteins in solution (Rg) were evaluated as a measure of the protein's overall compactness and mass distribution around its center of gravity [41], with results shown in Figure 9a.

The Rg analysis indicates that ligand binding results in minor adjustments in protein compactness. The lovastatin‐bound complex shows a slight tendency toward a more compact arrangement, while the simvastatin‐bound complex exhibits a subtle tendency toward increased structural flexibility.

The probability density of the radius of gyration, shown in Figure 9b, illustrates the distribution of conformational states throughout the simulation. These observations reflect subtle differences in compactness and flexibility, consistent with modest conformational adjustments rather than large scale structural changes. This interpretation is supported by RMSD and SASA analyses, which together indicate that ligand binding induces minor conformational adaptations without compromising overall protein stability.

In general, the Rg analysis revealed that ligand binding led to measurable conformational changes, where simvastatin displayed moderate increased flexibility and lovastatin assumed a more compact structural arrangement. These trends, in combination with RMSD and SASA data, provide a consistent view of protein stability in the ligand‐bound forms [62].

#### 3.3.4 RMSF

**FIGURE 10:**
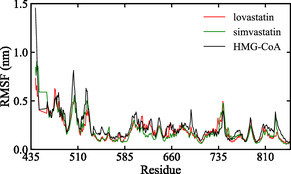
RMSF profiles of HMG‐CoA reductase in apo form and in complexes with lovastatin and simvastatin, illustrating decreased flexibility upon ligand binding.

The residue‐level flexibility of HMG‐CoA reductase was assessed and presented in Figure 10. The apo form exhibits elevated fluctuations, particularly at the N‐terminal region and near residues 500, 700, and 820, which correspond to regions involved in substrate binding, catalysis, and overall structural stability. These increased motions indicate greater conformational freedom in the absence of a bound ligand, suggesting that the protein structure is less stabilized without ligand interaction.

Upon complexation with lovastatin and simvastatin, a notable decrease in residue fluctuations is observed, suggesting ligand‐induced stabilization of the protein structure [63]. Among the two complexes, the simvastatin complex form shows the greatest reduction in flexibility across most residues. This enhanced stability can be attributed to the efficient binding of simvastatin within the active site, where hydrophobic interactions and hydrogen bonding networks likely restrict protein motions and promote structural rigidity.

Low flexibility in the simvastatin complex implies a stronger and more favorable interaction with the enzyme's catalytic site [64]. This increased stability is associated with improved inhibitory efficacy, as ligand‐induced stabilization often correlates with enhanced binding affinity and therapeutic potential.

#### 3.3.5 Hydrogen Bonding

**FIGURE 11:**
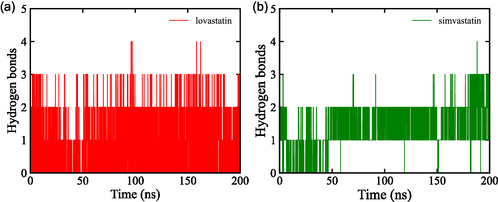
Hydrogen bond interactions between HMG‐CoA reductase and (a) lovastatin and (b) simvastatin during a 200 ns MD simulation, highlighting differences in binding stability.

In this study, the hydrogen bonding dynamics between statin derivatives and HMG‐CoA reductase were evaluated over a 200 ns simulation period. Lovastatin demonstrated a stable interaction profile during the simulation, consistently maintaining two to three hydrogen bonds with only slight fluctuations, as shown in Figure 11a. This stability can be attributed to the favorable positioning of its functional groups, which align well with hydrogen bond donors and acceptors in the active site, enabling persistent interactions [65]. In contrast, simvastatin displayed a less stable pattern, typically forming one to two hydrogen bonds on average but with frequent interruptions and short‐lived bonding events. This variability likely stems from its slightly bulkier side chain, which introduces steric hindrance and limits its ability to sustain stable hydrogen bonds, resulting in the more dynamic binding behavior observed, as shown in Figure 11b.

These observations suggest that lovastatin forms more stable hydrogen bonding networks with HMG‐CoA reductase, which correspond to stronger and more persistent binding affinity. Supporting literature [66, 67] indicates that ligands maintaining sustained hydrogen bonding throughout simulations are more likely to adopt stable binding conformations and demonstrate improved bioactivity. Therefore, the enhanced pharmacodynamic profile of lovastatin compared to simvastatin may be attributed, at least in part, to its greater hydrogen bond stability.

#### 3.3.6 Comparative Analysis of HMG‐CoA and its Complexes

**FIGURE 12:**
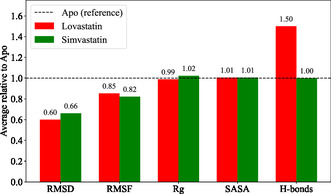
Normalized comparison of HMG‐CoA and its complexes, showing lovastatin's superior stabilization across RMSD, RMSF, Rg, SASA, and hydrogen bonds.

The comparative analysis of the MD simulation results demonstrates that lovastatin consistently stabilizes HMG‐CoA more effectively than simvastatin or the apo protein (Figure 12). When normalized relative to the apo protein (y = 1, unitless), lovastatin exhibits the lowest RMSD (0.36) and RMSF (0.20), indicating minimal global structural deviation and reduced residue‐level flexibility, particularly in the active site and surrounding secondary structural elements. This observation is consistent with the findings reported by Riyad et al, who similarly noted that stable ligand–protein interactions correspond to reduced atomic fluctuations and overall structural stability during MD simulation [68]. In contrast, simvastatin shows higher RMSD (0.40) and comparable RMSF (0.20), while the apo protein serves as the reference baseline (1.0), highlighting the stabilizing influence of ligand binding on protein dynamics. The radius of gyration (Rg) values further support this observation, with lovastatin maintaining a more compact protein conformation (2.55) relative to simvastatin (2.64) and apo (1.0), suggesting that its molecular structure promotes tighter packing through optimal hydrophobic interactions and π–π stacking within the active site. This trend is consistent with the findings of Arnedo, 2025 and Alruhaimi, 2025, who also reported that enhanced hydrophobic contacts and π–π stacking contribute significantly to protein structural compactness and conformational stability during MD simulations [69, 70].

Analysis of SASA indicates that lovastatin slightly increases surface exposure (206.45 nm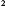, normalized to apo = 1.0), reflecting subtle conformational adjustments that may enhance solvent‐mediated stabilization. Notably, hydrogen bond analysis reveals that lovastatin forms a higher number of persistent interactions (3.2) with active‐site residues compared to simvastatin (2.8), underscoring stronger and more specific polar contacts that reinforce complex stability. This observation is consistent with the study of Fong, which showed that lovastatin maintained hydrogen bond stability with HMG‐CoA throughout the simulation [71]. Collectively, these normalized, unitless comparisons demonstrate that lovastatin achieves an optimal balance of structural stability, compactness, and specific molecular interactions, consistent with its superior binding affinity. The observed trends can be rationalized chemically: lovastatin's favorable stereochemistry and functional groups enable precise positioning within the active site, maximizing both enthalpic and entropic contributions to binding [72].

#### 3.3.7 FELs Analysis

**FIGURE 13:**
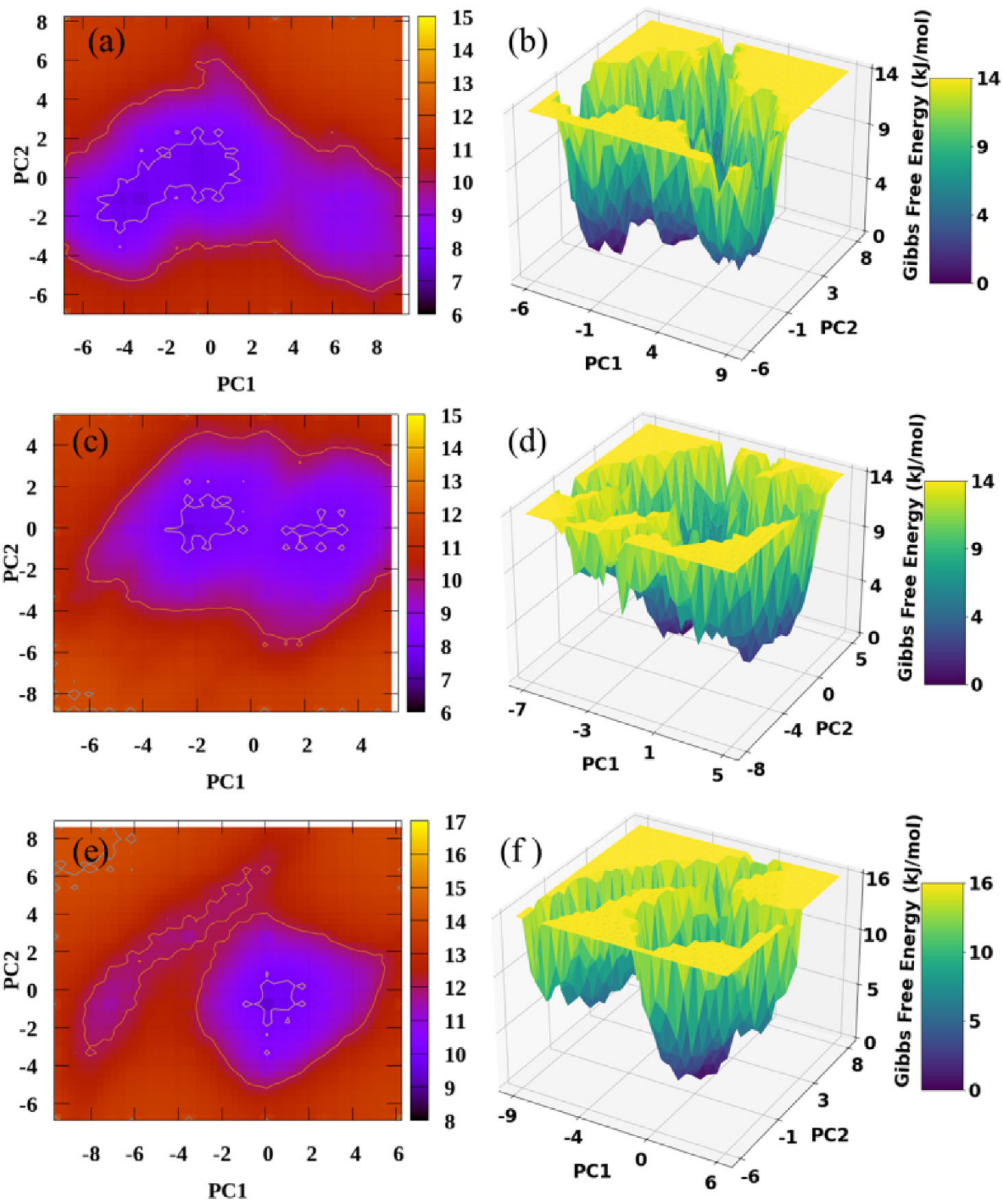
Conformational stability of apo HMG‐CoA reductase and its complexes with simvastatin and lovastatin, represented along PC1 and PC2. Energy basins indicate relative structural stability. Panels (a–b): Apo form; (c–d): Lovastatin complex; (e–f): Simvastatin complex.

The FELs of HMG‐CoA reductase in its apo form and in complex with lovastatin or simvastatin were analyzed and are presented in Figure 13. This analysis was interpreted alongside RMSD and hydrogen bonding trends to provide a comprehensive assessment of conformational stability.

The FEL for the apo HMG‐CoA reductase Figure 13a,b displays broad and multiple energy basins, reflecting high intrinsic flexibility in the absence of ligand binding. This conformational heterogeneity represents the dynamic nature of the protein and provides a reference for interpreting ligand‐induced effects.

In contrast, the HMG‐CoA–lovastatin complex (Figure 13c,d) forms a well‐defined dominant energy basin. The limited conformational fluctuations indicate that lovastatin restricts structural sampling, forming a well‐defined low‐energy basin. This observation is consistent with RMSD and hydrogen bonding analyses, indicating stabilization of the protein structure.

The HMG‐CoA–simvastatin complex (Figure 13e,f) exhibits a dominant basin and a secondary shallower basin, indicating moderate conformational flexibility compared with lovastatin. This reflects ligand‐dependent differences in protein dynamics, with simvastatin allowing broader structural sampling.

Overall, these FEL analyses demonstrate that ligand binding differentially modulates the protein's conformational landscape: apo HMG‐CoA explores multiple basins, lovastatin forms a well‐defined low‐energy basin, and simvastatin occupies intermediate states with moderate flexibility. These qualitative trends, supported by RMSD and hydrogen bonding analyses, provide a consistent and cautious interpretation of protein stability and dynamics.

## 4 Conclusion

This study used a thorough in silico approach to assess the pharmacokinetic, toxicological, and molecular interaction profiles of statin analogs that target HMG‐CoA reductase, a crucial enzyme in the production of cholesterol. Our work goes beyond routine screening by providing a mechanistic explanation for the superior therapeutic potential of statin analogs, thereby addressing the current gap in the literature.

From ADMET predictions, analogs of pravastatin, lovastatin, and simvastatin were identified as having favorable pharmacological profiles, with low anticipated toxicity, limited CYP450 enzyme inhibition, and good gastrointestinal absorption. Molecular docking further highlighted the strong binding affinities of the simvastatin and lovastatin analogs.

Crucially, our MD simulations provided the key insight into the superior behavior of these compounds. We demonstrated that the apo protein exists in multiple flexible conformations, whereas the binding of lovastatin and simvastatin analogs induces a significant stabilizing effect. This was evidenced by the reduction in structural fluctuations (RMSD and RMSF) and the confinement of the protein‐ligand complexes to a single, stable, low‐energy state, as shown by our FEL analysis. This ligand‐induced conformational rigidity is a powerful indicator of enhanced inhibitory efficacy and long‐term complex stability. The slightly lower radius of gyration (Rg) and prolonged hydrogen bonding observed with the lovastatin complex further emphasize its ability to promote a more compact and stable binding conformation.

In summary, these results collectively highlight the therapeutic potential of lovastatin and simvastatin analogs as potent HMG‐CoA reductase inhibitors. Their ability to not only bind to the target but also to mechanistically stabilize the enzyme in a favorable conformation sets them apart from conventional statin studies. This work offers a compelling new direction for the rational design of effective and stable treatments for CAD. Future research should focus on the experimental validation of these findings and structural optimization strategies aimed at enhancing potency and minimizing off‐target effects.

## Author Contributions

**Yoshua B. Mtulo:** investigation, conceptualization, data curation, formal analysis, methodology, software, validation, visualization, writing original draft, writing – review and editing. **Geradius Deogratias:** visualization, validation, data curation, writing ‐ review and editing. **James E. Mgaya:** supervision, project administration, conceptualization, investigation, methodology, validation, writing – review and editing. **Andrew S. Paluch:** supervision, funding acquisition, investigation, writing – review and editing. **Lucas Paul:** supervision, conceptualization, investigation, methodology, data curation, formal analysis, writing – review and editing.

## Conflicts of Interest

The authors declare no conflicts of interest.

## Data Availability

The data that support the findings of this study are available from the corresponding author upon reasonable request. We provide sample input files to reproduce the results of the present study on Zenodo at https://doi.org/10.5281/zenodo.17246303.
